# Gate‐Tunable Polarity‐Reconfigurable Photovoltaic Response With Dual Linearity in NbS_3_/WSe_2_ Heterostructures for In‐Sensor Image Processing

**DOI:** 10.1002/advs.77288

**Published:** 2026-09-06

**Authors:** Jidong Liu, Jianxiong Xie, Qiaoyan Hao, Yutao Hu, Zhen He, Haolin Liu, Danting Lin, Haibo Gan, Yudi Tu, Wenjing Zhang

**Affiliations:** ^1^ State Key Laboratory of Radio Frequency Heterogeneous Integration International Collaborative Laboratory of 2D Materials for Optoelectronics Science and Technology of Ministry of Education Institute of Microscale Optoelectronics Shenzhen University Shenzhen China

**Keywords:** bipolar photovoltaic response, dual linearity, gate‐tunable, in‐sensor computing, van der Waals heterostructure

## Abstract

Reconfigurable optoelectronic image sensors capable of in‐sensor computing are promising for energy‐efficient edge visual perception. Self‐powered photovoltaic operation further reduces the power demand of image sensing and processing. However, it remains challenging to achieve a continuously gate‐tunable self‐powered photovoltaic response that combines reversible polarity with linear weight programmability. Here, we demonstrate a gate‐tunable photovoltaic detector based on an NbS_3_/WSe_2_ van der Waals heterostructure. Owing to the ambipolar transport of WSe_2_ and the distinct electrostatic responses of the two constituent materials, gate voltage continuously modulates the interfacial band alignment and reversibly switches the built‐in electric field at the heterointerface. Consequently, the device exhibits gate‐controlled positive and negative photovoltaic responses. The short‐circuit photocurrent scales linearly with incident power density, while the short‐circuit photoresponsivity varies linearly with gate voltage over a broad programming window. This dual linearity allows the photoresponsivity to function as a programmable analog weight for in‐sensor convolution. As a proof of concept, several image convolution kernels are implemented for in‐sensor image processing, and the experimentally obtained outputs agree well with algorithmic simulations. This work provides a promising device‐level building block for reconfigurable and energy‐efficient vision sensors for edge computing.

## Graphical abstract

An NbS_3_/WSe_2_ van der Waals heterostructure realizes a continuously gate‐tunable self‐powered photovoltaic response through interfacial band‐alignment modulation and reversible built‐in electric‐field switching. The short‐circuit photocurrent scales linearly with incident power density, while short‐circuit photoresponsivity varies linearly with gate voltage. This dual linearity enables programmable analog weights for reconfigurable in‐sensor convolutional image processing.

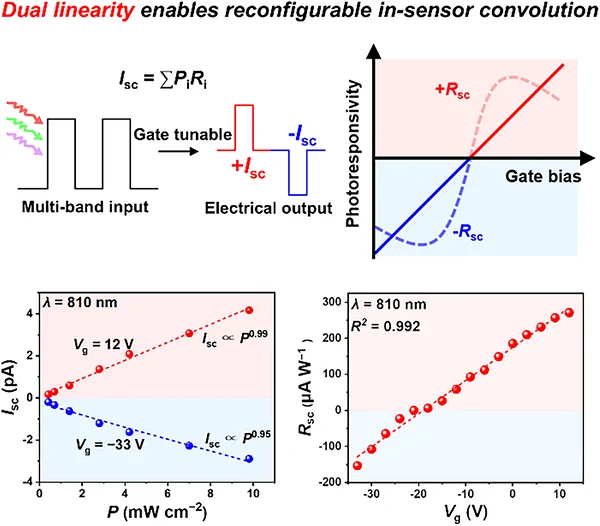

## 1 Introduction

Rapid advances in edge computing and the internet of things are driving increasing demand for intelligent vision systems that combine low power consumption with real‐time operation [1, 2, 3]. In conventional von Neumann architectures, image sensing and data processing are physically separated, resulting in substantial energy consumption, latency, and hardware redundancy caused by frequent data transfer and signal conversion between the sensor and the processor [4, 5]. By contrast, in‐sensor computing moves part or all of the computational workload to the sensing front end, allowing image acquisition and preliminary processing to occur within the same hardware platform [6, 7, 8]. This close integration of sensing and computation can substantially reduce redundant data movement and back‐end processing overhead, making it attractive for next‐generation machine vision, autonomous driving, and remote sensing applications [9, 10, 11, 12, 13, 14].

A central challenge in implementing in‐sensor computing is the development of optoelectronic devices that combine reliable photodetection capability with programmable response characteristics [15, 16]. In particular, such devices should provide sufficient sensitivity and fast response, while enabling linear and reversible modulation of photoresponsivity (*R*) across both positive and negative values. This capability permits hardware‐level emulation of positive and negative weights in neural network operations and supports a broad range of computational functions [17, 18]. Among the available modulation strategies, gate voltage control is especially attractive because it is compatible with standard semiconductor processes and offers precise electrical programmability [19, 20, 21]. However, conventional semiconductor photodetectors are constrained by fixed band structures and doping profiles, which prevent dynamic reconfiguration of the built‐in electric field and photocurrent direction and thereby limit their utility in reconfigurable in‐sensor computing [22]. To this end, research has shifted from fixed‐response photodetectors toward reconfigurable devices that enable electrical programming of sensing and computing functions. Self‐powered operation with linearly programmable bipolar photoresponses is especially attractive for low‐power machine vision, as it allows optical sensing and signed analog weighting to be integrated at the sensing front end. Yet achieving both reversible photovoltaic polarity and linear weight programmability in a single device is a critical prerequisite for building high‐fidelity in‐sensor computing systems.

Two‐dimensional (2D) van der Waals heterostructures provide a versatile materials platform for reconfigurable optoelectronic devices [23, 24, 25, 26]. Their atomic‐scale thickness enables the carrier type and concentration to be effectively modulated by gate bias, thereby shifting the Fermi level [27, 28]. When one constituent layer, such as an ambipolar semiconductor, exhibits a gate‐sensitive Fermi level while the other layer remains comparatively insensitive to the gate field, the relative Fermi‐level alignment between the two materials can be continuously tuned. This dynamic band engineering modifies the interfacial band bending and built‐in electric field, enabling reversible control over photocurrent polarity [29, 30]. Nevertheless, polarity switching alone is insufficient for accurate in‐sensor computing. The photoresponsivity also needs to vary linearly over a wide gate voltage range, because linearity is critical for precise and predictable weight programming through gate bias and for constructing reconfigurable convolution kernels [31, 32]. Recent 2D material‐based optoelectronic devices have enabled reconfigurable visual processing through different mechanisms, including gate‐tunable van der Waals heterostructures for positive and negative photoresponses and convolution [19, 31, 33], electrically programmed non‐volatile neuromorphic photovoltaics and photomemristors for stored responsivity states [16, 34], and persistent‐photoconductivity‐based heterostructures for mid‐infrared trajectory perception [35]. These advances demonstrate diverse routes toward the integration of sensing, memory, and computing, while also highlighting the importance of predictable electrical programming of photovoltaic polarity and analog photoresponsivity for accurate in‐sensor operations.

Here, we demonstrate a linearly reconfigurable photovoltaic detector based on an NbS_3_/WSe_2_ van der Waals heterostructure. This combination was chosen because WSe_2_ is ambipolar with strong gate dependence, whereas NbS_3_ maintains n‐type transport over the measured gate range. Their distinct electrostatic responses enable gate voltage to continuously tune the Fermi‐level alignment and even reverse the built‐in electric field, providing the physical basis for the polarity‐reconfigurable photovoltaic response. Optoelectronic measurements reveal reversible switching between positive and negative photovoltaic responses over a broad spectral range, while the short‐circuit photocurrent scales linearly with incident optical power. More importantly, the photoresponsivity varies linearly with gate voltage over a wide 45 V window, providing a basis for precise and programmable analog weights. Building on this dual linearity, we further demonstrate in‐sensor convolution for several image processing functions. Overall, this work experimentally validates a route toward linearly tunable bipolar photovoltaic response through gate‐controlled band engineering and suggests a promising strategy for reconfigurable and energy‐efficient in‐sensor image processing.

## 2 Results and Discussion

**FIGURE 1:**
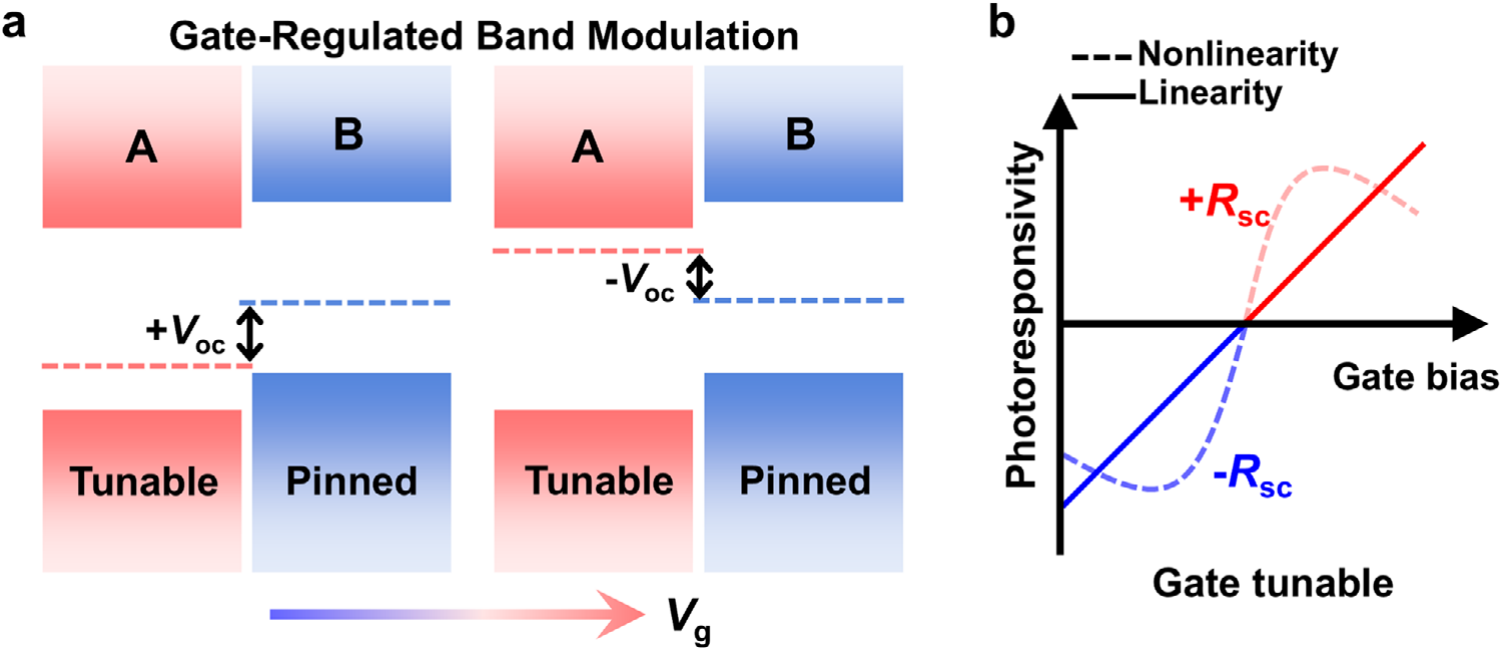
(a) Schematic illustration of band‐alignment modulation in the heterostructure under gate bias, enabling polarity‐reconfigurable photovoltaic response. (b) Comparison between the desired linear (solid line) and typical nonlinear (dashed line) relationships between gate‐tunable photoresponsivity and gate voltage.

Figure 1a summarizes a simplified materials‐selection rationale for polarity‐reconfigurable photovoltaic heterostructures. Material A possesses a strongly gate‐sensitive Fermi level, whereas material B exhibits a comparatively weaker electrostatic response, such that sufficient gate modulation can reverse their relative Fermi‐level alignment and consequently the interfacial band bending and built‐in electric field. Under a sufficiently negative gate bias, the Fermi level of material A shifts downward and the channel exhibits p‐type transport. The resulting Fermi level offset relative to material B generates a built‐in electric field (*E*_bi_) directed from material B to material A. Under illumination, this field separates photogenerated electron‐hole pairs and produces a negative photovoltaic current. As the gate voltage is increased, the Fermi level of material A shifts upward and the channel becomes n‐type. Consequently, the sign of the Fermi level offset is reversed, which reverses the built‐in electric field and gives rise to a positive photovoltaic current [36, 37]. Thus, by exploiting gate‐tunable band alignment and the different electrostatic responses of the two constituent materials, the interfacial band bending and built‐in electric field can be reversibly controlled, providing the physical basis for bipolar photovoltaic response with both positive and negative polarities [38, 39, 40]. In an in‐sensor computing architecture, the device photoresponsivity directly serves as the programmable weight in convolution operations [33, 41]. The mapping between weight and gate voltage therefore directly determines computational precision and trainability. The solid line in Figure 1b represents the ideal linear response required for in‐sensor computing, in which photoresponsivity changes linearly with gate voltage across both positive and negative values. Such a relationship allows both the sign and magnitude of the weight to be accurately set by gate bias, supporting reliable convolution operations. By contrast, the dashed line represents a more common nonlinear response. Although the photoresponsivity can still switch from negative to positive values, such nonlinear mapping makes the programmed weight less predictable and less precise. This nonlinearity can introduce computational errors and consequently limit the use of such devices in applications that require accurate weight updates and high‐fidelity image processing [31, 32].

**FIGURE 2:**
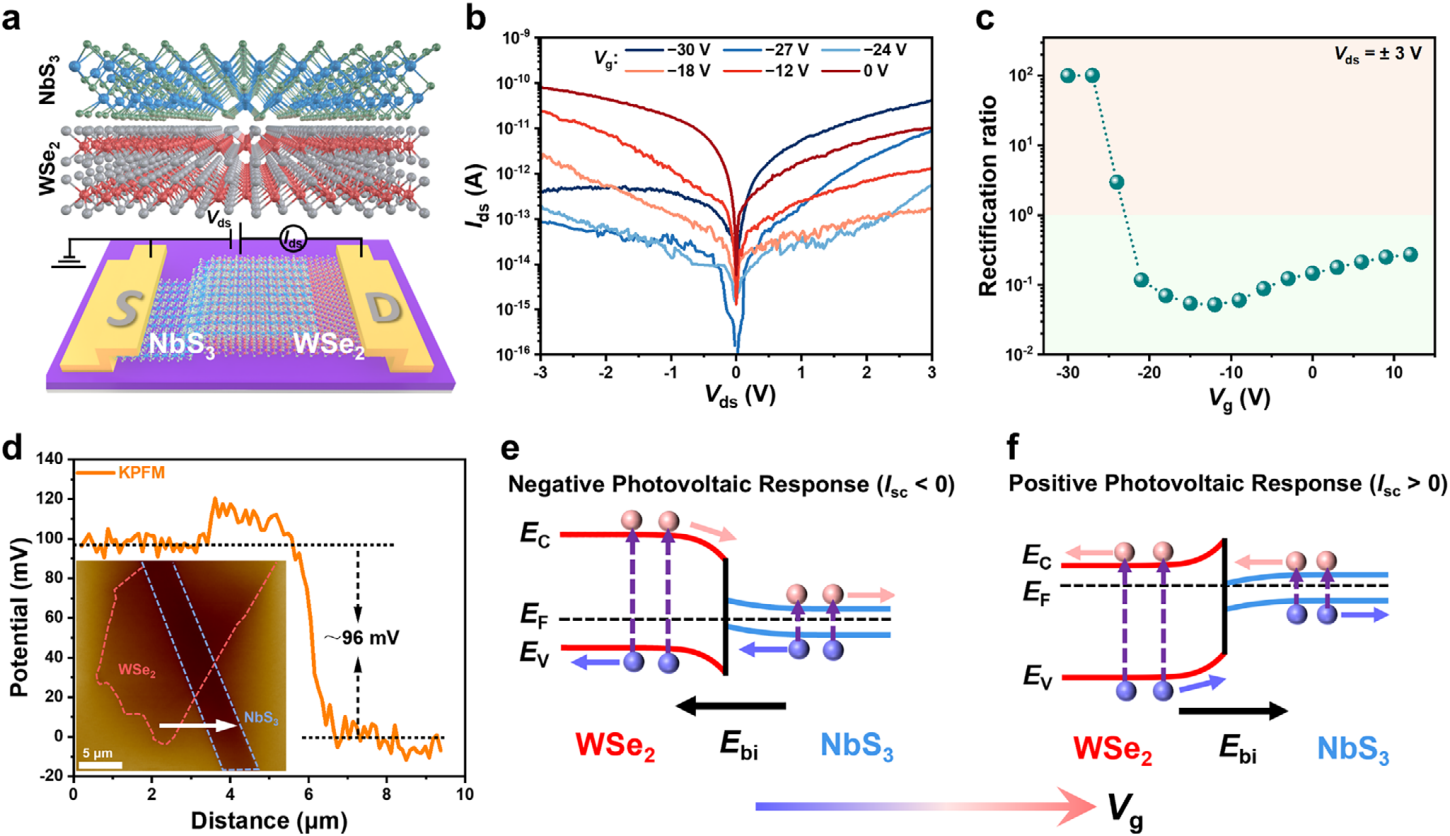
(a) Schematic diagram of the NbS_3_/WSe_2_ heterostructure. (b) Output curves under gate voltage modulation, showing gate‐reconfigurable bidirectional rectification. (c) Rectification ratio (|*I*_forward_| / |*I*_reverse_|) as a function of *V*_g_ at *V*_ds_ = ±3 V. (d) KPFM image of the NbS_3_/WSe_2_ heterostructure and surface potential profile extracted along the white arrow. Schematic band alignments of the NbS_3_/WSe_2_ heterostructure in representative gate regimes corresponding to (e) negative and (f) positive photovoltaic responses.

Figure 2a schematically shows the NbS_3_/WSe_2_ heterostructure fabricated on a SiO_2_/Si substrate. The electrode connected to WSe_2_ is defined as the drain, the grounded electrode connected to NbS_3_ is defined as the source, and the bottom Si substrate serves as the gate. Raman spectroscopy with 532 nm excitation was used to verify heterostructure formation and the crystalline quality of the constituent materials. As shown in Figure S1, Raman spectra collected from the individual flakes and the overlap region exhibit the characteristic peaks of NbS_3_ and WSe_2_, confirming successful assembly of the heterostructure [42, 43, 44]. The transfer characteristics of individual NbS_3_ and WSe_2_ devices are provided in Figure S2, showing that NbS_3_ behaves as an n‐type semiconductor, whereas WSe_2_ exhibits ambipolar transport with strong gate dependence. The gate‐modulated output characteristics of the heterostructure are presented in Figure 2b and show typical diode‐like behavior. Notably, the rectification direction reverses under external gate modulation, consistent with the ambipolar nature of the WSe_2_ channel. The rectification ratio extracted as a function of *V*_g_ is summarized in Figure 2c. Here, the rectification ratio is defined as |*I*_ds_(+3 V)| / |*I*_ds_(−3 V)|, where the absolute current values are used to compare the relative conduction asymmetry under opposite *V*_ds_. As *V*_g_ is swept from positive to negative values, the rectification ratio evolves from below unity to above unity, indicating a gate‐induced reversal of the dominant rectification direction. This gate‐reconfigurable rectification provides electrical evidence that interfacial band bending and built‐in electric field can be continuously modulated by electrostatic gating [45, 46].

To further probe interfacial carrier transfer and band alignment in the NbS_3_/WSe_2_ heterostructure, Kelvin probe force microscopy (KPFM) was performed to map the surface potential distribution across the heterointerface. The atomic force microscopy (AFM) topography and corresponding height profiles are provided in Figure S3. Owing to the different work functions of the two constituent layers, the KPFM image in Figure 2d reveals clear potential steps across the heterointerfaces. The contact potential difference (CPD) between the AFM tip and the material surface is defined as CPD_material_ = (*Ф*_tip_—*Ф*_material_)/*e*, where *e* is the elementary charge and *Ф*_tip_ and *Ф*_material_ are the work functions of the tip and the material, respectively [44, 47]. The line profile extracted from Figure 2d indicates that the Fermi level of WSe_2_ is approximately 96 meV higher than that of NbS_3_. The corresponding band alignment between NbS_3_ and WSe_2_ is provided in Figure S4. Based on these results, the mechanisms for gate‐reconfigurable photovoltaic response are schematically illustrated in Figure 2e,f. Under sufficiently negative gate bias, the heterostructure can be approximately described as n‐type NbS_3_ coupled with hole‐dominated WSe_2_. This interpretation is supported by the transfer characteristics in Figure S2, where WSe_2_ exhibits ambipolar transport with hole‐dominated conduction at negative *V*_g_, whereas NbS_3_ retains n‐type transport throughout the measured gate‐voltage range. In this representative negative‐response regime, the interfacial built‐in electric field drives photogenerated electrons toward NbS_3_ and holes in the opposite direction under illumination, resulting in a negative short‐circuit photocurrent. As *V*_g_ is increased, the Fermi level of WSe_2_ shifts upward, and the WSe_2_ channel becomes increasingly electron‐dominated. The heterostructure therefore gradually evolves toward an n‐type NbS_3_/electron‐dominated WSe_2_ configuration. In the representative positive‐response regime, the change in relative band alignment reverses the interfacial built‐in electric field and the direction of carrier separation, leading to a positive short‐circuit photocurrent.

**FIGURE 3:**
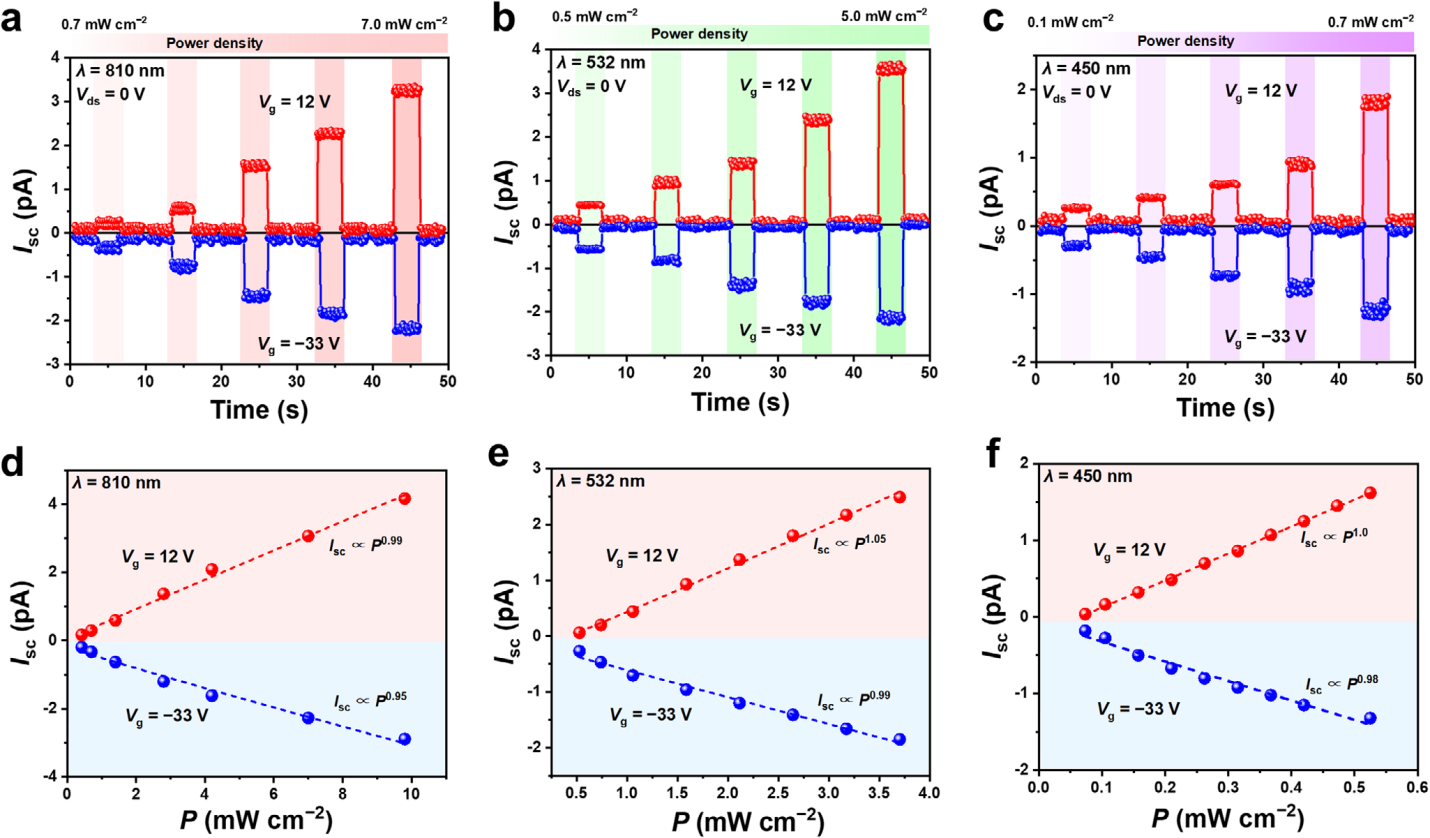
Positive and negative photoresponses under varying power densities (indicated by different shading levels) at *V*_g_ = 12 V and *V*_g_ = −33 V under illumination at (a) 810 nm, (b) 532 nm, and (c) 450 nm. Extracted *I*_sc_ as a function of power density at *V*_g_ = 12 V and *V*_g_ = −33 V under illumination at (d) 810 nm, (e) 532 nm, and (f) 450 nm.

The transport characteristics described above indicate that gate bias effectively modulates the built‐in electric field in the NbS_3_/WSe_2_ heterostructure. We therefore investigated the gate‐controlled photovoltaic characteristics of the NbS_3_/WSe_2_ photodetector. The left panel of Figure S5a shows the *I*_ds_‐*V*_ds_ curves measured under 810 nm illumination at *V*_g_ = 12 V for different power densities. Under these conditions, the device exhibits a positive short‐circuit current (*I*_sc_) and a negative open‐circuit voltage (*V*_oc_). For clarity, we use the sign of *I*_sc_ to distinguish positive and negative photovoltaic responses. This operating condition is therefore defined as the positive photovoltaic response. By contrast, the *I*_ds_‐*V*_ds_ curves measured at *V*_g_ = −33 V under 810 nm illumination show a negative *I*_sc_ and a positive *V*_oc_, corresponding to a negative photovoltaic response (right panel of Figure S5a). Similar gate‐dependent switching between positive and negative photovoltaic responses is observed under 532 and 450 nm illumination, as shown in Figure S5, indicating that polarity‐reconfigurable photovoltaic behavior is maintained over a broad spectral range. Figure 3a–c show the time‐resolved photoresponse of the NbS_3_/WSe_2_ photodetector under 810, 532, and 450 nm illumination at *V*_g_ = 12 V and *V*_g_ = −33 V, with different shading levels representing different power densities. At each wavelength, the photodetector exhibits gate‐tunable positive and negative photocurrents, and the magnitude of both responses increases with power density. The extracted *I*_sc_ values as a function of power density under different wavelengths at *V*_g_ = 12 V and *V*_g_ = −33 V are presented in Figure 3d–f. The power‐dependent *I*_sc_ was fitted using *I*_sc_ ∝ *P^α^*, where *P* is the incident power density. The fitted *α* values are close to 1 for all tested wavelengths, indicating an approximately linear relationship between *I*_sc_ and incident power density for both positive and negative photovoltaic responses [18, 36]. Such near‐linear behavior is favorable for high‐fidelity image information acquisition.

To clarify the origin of the photovoltaic response, spatially resolved photocurrent mapping was performed under different gate voltages. As shown in Figure S6, positive photocurrent is predominantly localized in the NbS_3_/WSe_2_ overlap region at *V*_g_ = 12 V, whereas the photocurrent in the same region reverses to negative polarity at *V*_g_ = −30 V. No significant photoresponse is observed at the metal electrodes or graphene/semiconductor contact areas, supporting that the gate‐reconfigurable photovoltaic response is dominated by the gate‐modulated built‐in electric field at the NbS_3_/WSe_2_ heterointerface [48, 49]. Figure S7 shows the response speeds of the positive and negative photovoltaic modes under laser irradiation. The rise and fall times are 349 and 376 µs, respectively, for the positive photoresponse, and 418 and 457 µs, respectively, for the negative photoresponse. This fast response supports the use of the NbS_3_/WSe_2_ photodetector for rapid image processing applications. Furthermore, we evaluated the cyclic stability of the positive and negative photovoltaic modes of the NbS_3_/WSe_2_ heterostructure device at *V*_g_ = 12 V and *V*_g_ = −33 V, respectively. As shown in Figure S8, both modes maintain their respective polarity and stable response amplitudes under repeated 810, 532, and 450 nm illumination without obvious degradation over the measured cycles, confirming good reproducibility over the tested spectral range. Since the corresponding *R*_sc_ values serve as programmable analog weights for convolution, stable photoresponse is essential for preserving both the sign and magnitude of the weights. Variations in the photoresponse would cause deviations in the programmed weights and consequently reduce the reproducibility and fidelity of the processed images. The observed cyclic stability therefore supports the reliable use of the programmed photovoltaic responses for in‐sensor image processing.

**FIGURE 4:**
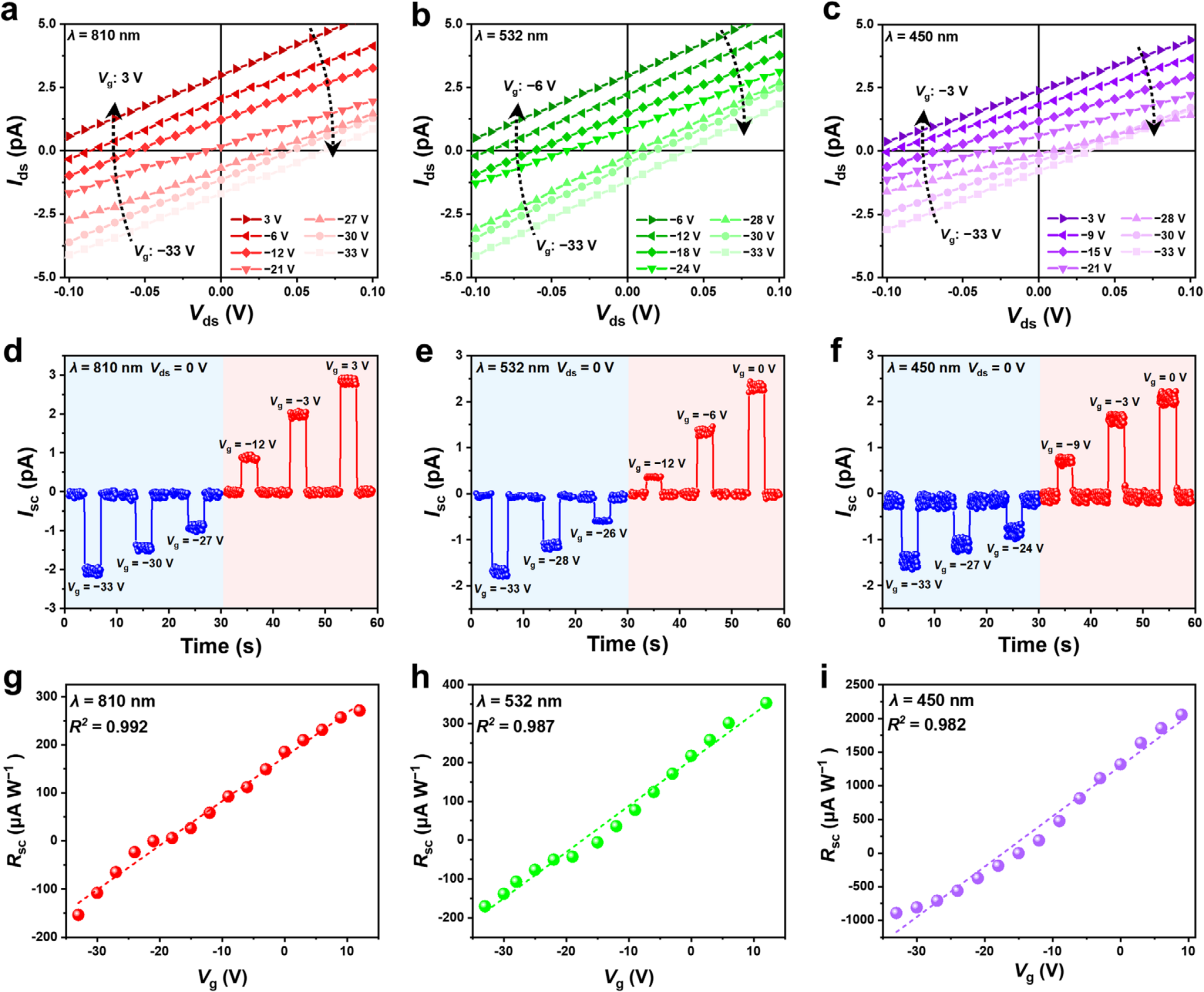
*I*_ds_‐*V*_ds_ curves measured at different *V*_g_ values under illumination at (a) 810 nm, (b) 532 nm, and (c) 450 nm. Gate‐tunable switching between negative and positive photovoltaic responses under illumination at (d) 810 nm, (e) 532 nm, and (f) 450 nm. Calculated *R*_sc_ as a function of *V*_g_ from −33 to 12 V under illumination at (g) 810 nm, (h) 532 nm, and (i) 450 nm.

To evaluate the continuous gate tunability of the reconfigurable photovoltaic response, we performed gate voltage‐dependent optoelectronic measurements under 810, 532, and 450 nm illumination. Figure 4a shows the *I*_ds_‐*V*_ds_ curves measured under 810 nm illumination. Over the gate voltage range from −33 to 3 V, a reversible transition in the photovoltaic response is clearly observed. At *V*_g_ = −33 V, the heterostructure exhibits a negative *I*_sc_, whereas the current polarity becomes positive as *V*_g_ is gradually increased to 3 V. This result confirms that the photovoltaic polarity of the NbS_3_/WSe_2_ heterostructure can be reversibly reconfigured by gate bias. Similar behavior is observed under 532 and 450 nm illumination, demonstrating that the gate‐tunable photovoltaic response is robust across a broad spectral range (Figure 4b,c). Under fixed power density, we further measured the time‐resolved photoresponse at different gate voltages for 810, 532, and 450 nm illumination. As shown in Figure 4d–f, distinct positive and negative photocurrents are observed at zero bias across the continuously tunable gate voltage range, in agreement with the gate‐dependent *I*_ds_‐*V*_ds_ characteristics. These results further verify that the photovoltaic current polarity can be reversibly programmed through electrostatic gating. A similar gate‐tunable switching behavior between negative and positive zero‐bias photocurrents is also observed in another independently fabricated NbS_3_/WSe_2_ heterostructure device (Figure S9). Beyond the linear mapping between optical power and photocurrent, in‐sensor convolution also requires a linear mapping between gate voltage and photoresponsivity. We therefore extracted the gate‐dependent short‐circuit photoresponsivity (*R*_sc_) at different wavelengths. As shown in Figure 4g–i, the photovoltaic current measured over the gate voltage range from −33 to 12 V was converted into the corresponding positive and negative *R*_sc_ values. Notably, *R*_sc_ exhibits a near‐linear dependence on gate voltage throughout the programmed window under all tested illumination wavelengths. Linear fitting of the *R*_sc_‐*V*_g_ curves yields *R* ^2^ values of 0.992, 0.987, and 0.982 under 810, 532, and 450 nm illumination, respectively, indicating near‐linear gate programmability of the photoresponsivity. While residual nonlinearity may introduce finite errors in the programmed analog weights and the convolution output, the high fitting coefficients confirm that these deviations are limited over the investigated programming range. For more general convolution operations, the programming accuracy can be further enhanced by calibrating the *R*_sc_‐*V*_g_ relationship and applying a compensated voltage‐to‐weight mapping. This 45 V gate‐voltage window enables direct programming of both the sign and magnitude of the convolution weight, thereby supporting the construction of different convolution kernels for multiple image processing functions. In future devices, the required gate‐voltage range could be reduced by improving the gate‐coupling efficiency through thinner dielectrics, high‐*κ* gate insulators, or local‐gate architectures. Furthermore, the photoresponsivity could be enhanced through optical cavities or plasmonic structures to improve light absorption, together with interface passivation or contact engineering to reduce recombination and collection losses, while preserving the linear *I*_sc_‐*P* and *R*_sc_‐*V*_g_ relationships.

**FIGURE 5:**
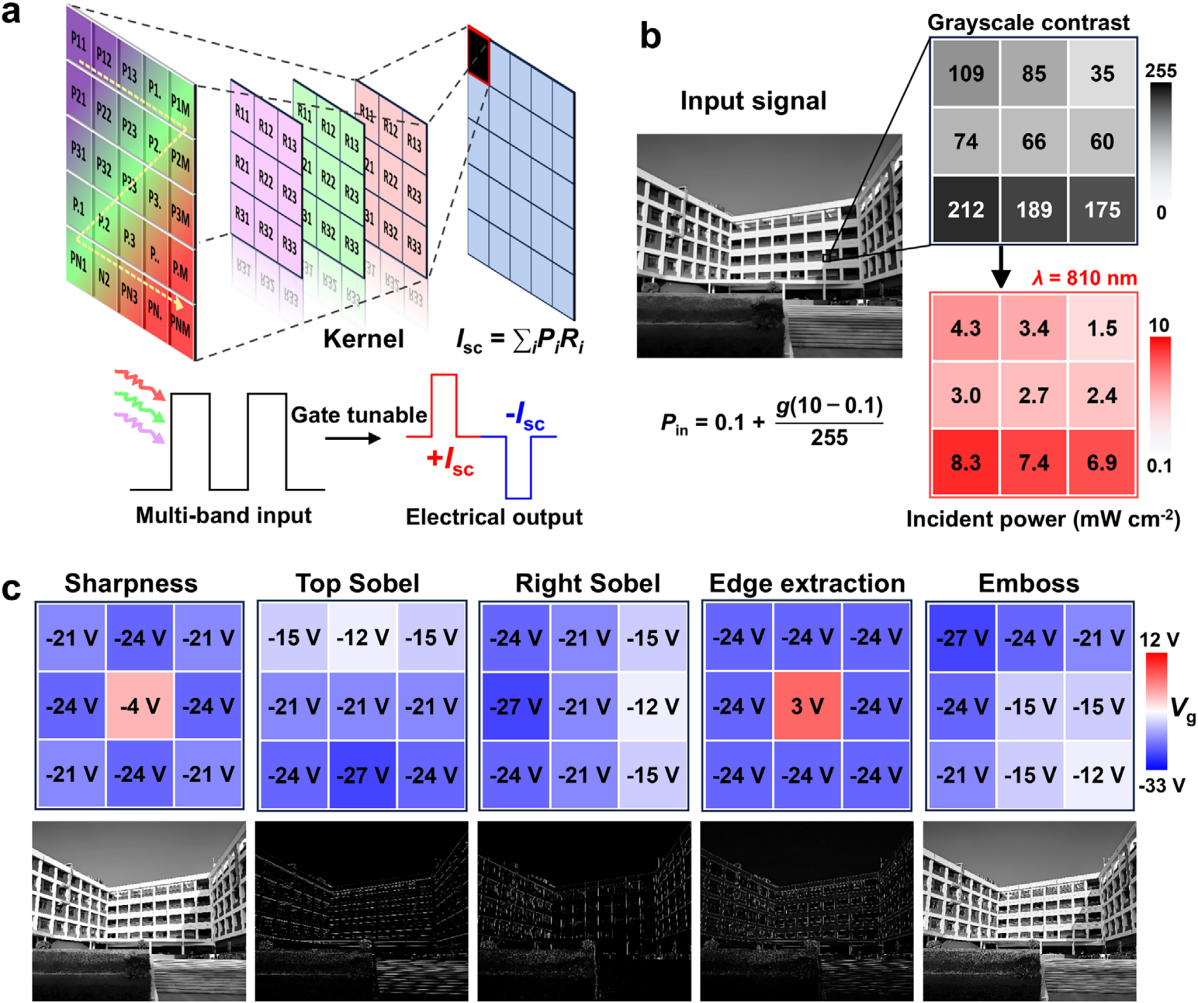
(a) Schematic illustration of in‐sensor image processing using gate‐tunable switching between negative and positive photocurrent in the NbS_3_/WSe_2_ heterostructure device. The input signal, represented by an optical signal under laser illumination, is processed by a 3 × 3 reconfigurable matrix kernel using *V*_g_ modulation to generate negative and positive *R*_sc_ values. (b) The input image is a 256 × 256 grayscale image (left). A representative 3 × 3 pixel region (top, right) from the input image is converted from grayscale to incident power (bottom, right). The inset shows the equation used to convert the input image from an 8‐bit grayscale map to an incident power map. (c) Demonstration of in‐sensor convolutional image processing using experimentally reconfigurable kernels, including sharpness, top Sobel, right Sobel, edge extraction, and emboss. The upper panel shows the gate‐voltage matrices used to program the experimental kernels, and the lower panel shows the corresponding processed images.

Gate voltages of different polarities effectively modulate the built‐in electric field of the NbS_3_/WSe_2_ heterostructure photodetector, producing reconfigurable positive and negative photovoltaic responses. Because the *I*_sc_ scales linearly with power density and the *R*_sc_ varies linearly with gate voltage, the device can perform simultaneous optical sensing and convolution operations. Figure 5a schematically illustrates the in‐sensor image processing mechanism based on the gate‐tunable heterostructure photodetector. As a proof of concept, a single NbS_3_/WSe_2_ device was operated sequentially to realize the nine elements of each 3 × 3 convolution kernel. For each image patch, pixel intensities were encoded as incident power densities *P*_i_, and the gate voltage was sequentially adjusted to program the required photoresponsivities *R*_i_. The nine photocurrent contributions were measured sequentially and summed as *I*_sc_ = ∑*P*_i_ *R*_i_ to yield one convolution output [50, 51]. This demonstrates single‐device sequential convolution, rather than simultaneous operation of a physical array. A future parallel implementation would require independently gated detectors with their photocurrents summed on a common output line (Figure S10).

We then carried out proof‐of‐concept convolution image processing under 810 nm illumination. Each grayscale value in the input image (0–255) was converted into a corresponding incident power density *P*_i_, ranging from 0.1 to 10 mW cm^−^^2^. As shown in Figure 5b, the upper 3 × 3 panel corresponds to the grayscale values of the original image, whereas the lower 3 × 3 panel represents the corresponding *P*_i_ values. Figure 5c presents the convolutional image processing results for several filters, including sharpness, top Sobel, right Sobel, edge extraction, and emboss, using the reconfigurable convolution kernel programmed in the NbS_3_/WSe_2_ device. The upper panel displays the gate‐voltage matrices used to program the experimental convolution kernels, and the lower panel shows the corresponding processed images obtained using these kernels. Similar in‐sensor convolution operations can also be performed under 532 and 450 nm illumination, as shown in Figures S11 and S12. Comparison with algorithmic simulations using standard kernels (Figure S13) shows that the experimentally programmed kernels reproduce the key image features. These results validate the feasibility of using the gate‐programmable positive and negative photoresponsivity of NbS_3_/WSe_2_ heterostructures as analog convolution weights, while future array integration will be required for fully parallel in‐sensor image processing.

## 3 Conclusion

In summary, we have demonstrated a gate‐tunable photovoltaic detector based on an NbS_3_/WSe_2_ van der Waals heterostructure with reconfigurable bipolar response. Through electrostatic modulation of the WSe_2_ Fermi level, the built‐in electric field at the heterointerface can be continuously reversed, enabling the photocurrent polarity to switch between negative and positive values under illumination. Optoelectronic characterization confirmed that the short‐circuit photocurrent scaled linearly with incident power density, while the photoresponsivity varied linearly with gate voltage over a 45 V programming window. This dual linearity enabled the photoresponsivity to function as a controllable analog weight in convolution operations, with the gate voltage directly defining both the sign and magnitude of the weight. We further demonstrated several image convolution operations using a single device, and the experimental results agreed well with algorithmic simulations. These results indicate that the NbS_3_/WSe_2_ heterostructure offers a viable platform for integrating sensing and computation within a single pixel element. Further development of device arrays and on‐chip training strategies may facilitate future in‐sensor processors for edge applications.

## 4 Experimental Section

### 4.1 Device Fabrication

Single‐crystalline WSe_2_ was commercially obtained from HQ Graphene, while NbS_3_ single crystals were grown according to our previous work [52]. A highly doped (100) p‐type silicon wafer with a 300 nm thermal oxide layer (Addison) was cut and used as the device substrate. The NbS_3_/WSe_2_ heterostructure was assembled through mechanical exfoliation and dry transfer. First, few‐layer WSe_2_ flakes were mechanically exfoliated directly onto a Si substrate with a 300 nm thermal oxide layer. An NbS_3_ flake was exfoliated onto a polydimethylsiloxane film supported on a glass slide, and then precisely aligned and transferred onto the target WSe_2_ flake using a home‐built micromanipulator system. The same transfer procedure was repeated to transfer graphene layers as contact electrodes at both ends of the NbS_3_/WSe_2_ heterostructure. To improve contact quality, the fabricated devices were annealed at 200°C for 2 h under an argon atmosphere.

### 4.2 Characterization and Photoresponse Measurements

Optical microscopy was performed using a Nikon Y‐IDP microscope. Raman spectroscopy and photocurrent mapping were conducted using a WITec Alpha 300R confocal microscope system equipped with a 100× or 50× objective and a 532 nm excitation laser. During photocurrent mapping, the current signal was amplified and recorded with a transimpedance amplifier, while an arbitrary function generator (DG5252, Rigol) was used to apply bias to the devices. Atomic force microscopy and Kelvin probe force microscopy measurements were carried out using a Bruker Dimension Icon system. The electrical and optoelectronic characteristics of the devices were measured at room temperature under high vacuum using a Keithley 4200 semiconductor parameter analyzer combined with a Lake Shore TTPX cryogenic probe station. A supercontinuum white light laser (SC400‐8, Fianium) coupled to a monochromator (AOTF‐V1, Fianium) was used as the light source, and the incident power was calibrated using a commercial Thorlabs power meter (PM200). For time‐resolved photoresponse characterization, the device output was connected to a Femto DLPCA‐200 amplifier and recorded using a Tektronix TDS2024C digital oscilloscope. A 520 nm diode laser (Coherent OBIS) served as the excitation source, modulated by a Rigol DG5252 function generator.

## Conflicts of Interest

The authors declare no conflicts of interest.

## Supporting information




**Supporting File 1**: advs77288‐sup‐0001‐SuppMat.docx.

## Data Availability

The data that support the findings of this study are available from the corresponding author upon reasonable request.

## References

[1] W. Shi , J. Cao , Q. Zhang , Y. Li , and L. Xu , “Edge Computing: Vision and Challenges,” IEEE Internet of Things Journal 3 (2016): 637–646, 10.1109/JIOT.2016.2579198.

[2] F. Zhou , Z. Zhou , J. Chen , et al., “Optoelectronic Resistive Random Access Memory for Neuromorphic Vision Sensors,” Nature Nanotechnology 14 (2019): 776–782, 10.1038/s41565-019-0501-3. 31308498

[3] K. Cui , S. Rao , S. Xu , et al., “Spectral Convolutional Neural Network Chip for In‐Sensor Edge Computing of Incoherent Natural Light,” Nature Communications 16 (2025): 81, 10.1038/s41467-024-55558-3. PMC11696300 39747892

[4] H. Wang , B. Sun , S. S. Ge , et al., “On Non‐Von Neumann Flexible Neuromorphic Vision Sensors,” npj Flexible Electronics 8 (2024): 28.

[5] H. Tang , N. Yu , P. Min , R. Guo , and G. Zhang , “In‐Sensor‐Memory Computing for Post‐Von Neumann Intelligence: A Perspective,” Nano‐Micro Letters 18 (2026): 338, 10.1007/s40820-026-02191-y. 42002681 PMC13092463

[6] F. Zhou and Y. Chai , “Near‐Sensor and In‐Sensor Computing,” Nature Electronics 3 (2020): 664–671, 10.1038/s41928-020-00501-9.

[7] Y. Chai , “In‐Sensor Computing for Machine Vision,” Nature 579 (2020): 32–33, 10.1038/d41586-020-00592-6. 32132685

[8] L. Mennel , J. Symonowicz , S. Wachter , D. K. Polyushkin , A. J. Molina‐Mendoza , and T. Mueller , “Ultrafast Machine Vision with 2D Material Neural Network Image Sensors,” Nature 579 (2020): 62–66, 10.1038/s41586-020-2038-x. 32132692

[9] P. Wu , T. He , H. Zhu , et al., “Next‐Generation Machine Vision Systems Incorporating Two‐Dimensional Materials: Progress and Perspectives,” InfoMat 4 (2022): 12275, 10.1002/inf2.12275.

[10] H. Jang , C. Liu , H. Hinton , et al., “An Atomically Thin Optoelectronic Machine Vision Processor,” Advanced Materials 32 (2020): 2002431, 10.1002/adma.202002431. 32700395

[11] F. Liao , Z. Zhou , B. J. Kim , et al., “Bioinspired In‐Sensor Visual Adaptation for Accurate Perception,” Nature Electronics 5 (2022): 84–91, 10.1038/s41928-022-00713-1.

[12] S. Liu , L. Liu , J. Tang , B. Yu , Y. Wang , and W. Shi , “Edge Computing for Autonomous Driving: Opportunities and Challenges,” Proceedings of the IEEE 107 (2019): 1697–1716, 10.1109/JPROC.2019.2915983.

[13] G. Feng , X. Zhang , B. Tian , and C. Duan , “Retinomorphic Hardware for In‐Sensor Computing,” InfoMat 5 (2023): 12473, 10.1002/inf2.12473.

[14] Y. Yu , M. Zhong , T. Xiong , et al., “Spectrometer‐Less Remote Sensing Image Classification Based on Gate‐Tunable van der Waals Heterostructures,” Advanced Science 11 (2024): 2309781, 10.1002/advs.202309781. 38610112 PMC11200008

[15] Q. Ren , C. Zhu , S. Ma , et al., “Optoelectronic Devices for In‐Sensor Computing,” Advanced Materials 37 (2025): 2407476, 10.1002/adma.202407476. 39004873 PMC12160704

[16] T. Li , J. Miao , X. Fu , et al., “Reconfigurable, Non‐Volatile Neuromorphic Photovoltaics,” Nature Nanotechnology 18 (2023): 1303–1310, 10.1038/s41565-023-01446-8. 37474683

[17] J. Han , W. Deng , F. Hu , et al., “2D Materials‐Based Photodetectors with Bi‐Directional Responses in Enabling Intelligent Optical Sensing,” Advanced Functional Materials 35 (2025): 2423360, 10.1002/adfm.202423360.

[18] G. Wu , X. Zhang , G. Feng , et al., “Ferroelectric‐Defined Reconfigurable Homojunctions for In‐Memory Sensing and Computing,” Nature Materials 22 (2023): 1499–1506, 10.1038/s41563-023-01676-0. 37770677

[19] C.‐Y. Wang , S.‐J. Liang , S. Wang , et al., “Gate‐tunable van der Waals Heterostructure for Reconfigurable Neural Network Vision Sensor,” Science Advances 6 (2020): aba6173, 10.1126/sciadv.aba6173. PMC7314516 32637614

[20] Y. Wang , H. Sun , Z. Sheng , et al., “Van der Waals Contacted WSe_2_ Ambipolar Transistor for In‐Sensor Computing,” Nano Research 16 (2023): 12713–12719, 10.1007/s12274-023-6128-6.

[21] H. Wang , Y. Li , P. Gao , et al., “Polarization‐ and Gate‐Tunable Optoelectronic Reverse in 2D Semimetal/Semiconductor Photovoltaic Heterostructure,” Advanced Materials 36 (2024): 2309371, 10.1002/adma.202309371. 37769436

[22] H. Jang , H. Hinton , W.‐B. Jung , et al., “In‐sensor Optoelectronic Computing Using Electrostatically Doped Silicon,” Nature Electronics 5 (2022): 519–525, 10.1038/s41928-022-00819-6.

[23] A. Castellanos‐Gomez , X. Duan , Z. Fei , et al., “Van der Waals Heterostructures,” Nature Reviews Methods Primers 2 (2022): 58, 10.1038/s43586-022-00139-1.

[24] Y. Meng , J. Feng , S. Han , et al., “Photonic van der Waals Integration From 2D Materials to 3D Nanomembranes,” Nature Reviews Materials 8 (2023): 498–517, 10.1038/s41578-023-00558-w.

[25] J. Liu , Y. Hu , H. Liu , et al., “Graphene‐Interlayered Sandwich Heterostructures With Step‐Like Band Alignment for Enhanced Electronic Transport and Fast Photodetection,” Nano Letters 26 (2026): 5045–5054, 10.1021/acs.nanolett.6c00073. 41958029

[26] S. Lee , R. Peng , C. Wu , and M. Li , “Programmable Black Phosphorus Image Sensor for Broadband Optoelectronic Edge Computing,” Nature Communications 13 (2022): 1485, 10.1038/s41467-022-29171-1. PMC8933397 35304489

[27] C. Pan , C.‐Y. Wang , S.‐J. Liang , et al., “Reconfigurable Logic and Neuromorphic Circuits Based on Electrically Tunable Two‐Dimensional Homojunctions,” Nature Electronics 3 (2020): 383–390, 10.1038/s41928-020-0433-9.

[28] R. Li , F. Lu , J. Deng , X. Fu , W. Wang , and H. Tian , “Bidirectional Rectifier With Gate Voltage Control Based on Bi_2_O_2_Se/WSe_2_ Heterojunction,” Journal of Semiconductors 45 (2024): 012701, 10.1088/1674-4926/45/1/012701.

[29] Y. Wang , J. Huang , Z. Pan , et al., “Reconfigurable Bidirectional Phototransistors Enabled by Graphene‐Integrated Asymmetric PtTe_2_/WSe_2_ Van der Waals Heterojunction,” Advanced Optical Materials 13 (2025): 02265, 10.1002/adom.202502265.

[30] H. Guo , Z. Gao , Z. Cheng , et al., “Giant Negative Photoconductivity in a Van Der Waals Heterojunction‐Based FET With Bidirectional Photoresponse,” Advanced Functional Materials 36 (2026): 28895, 10.1002/adfm.202528895.

[31] L. Pi , P. Wang , S.‐J. Liang , et al., “Broadband Convolutional Processing Using Band‐Alignment‐Tunable Heterostructures,” Nature Electronics 5 (2022): 248–254, 10.1038/s41928-022-00747-5.

[32] Z. Han , Y. Zhang , Q. Mi , et al., “Reconfigurable Homojunction Phototransistor for Near‐Zero Power Consumption Artificial Biomimetic Retina Function,” ACS Nano 18 (2024): 29968–29977, 10.1021/acsnano.4c10619. 39410794

[33] V. K. Dat , M. C. Nguyen , B. J. Jeong , et al., “Van der Waals Integration of 1D Nb_2_Pd_3_Se_8_ and 2D WSe_2_ for Gate‐Tunable In‐Sensor Image Processing,” Advanced Materials 37 (2025): 00011, 10.1002/adma.202500011. PMC12574645 40801229

[34] X. Fu , T. Li , B. Cai , et al., “Graphene/MoS_2−_ * _x_ *O* _x_ */Graphene Photomemristor With Tunable Non‐Volatile Responsivities for Neuromorphic Vision Processing,” Light: Science & Applications 12 (2023): 39, 10.1038/s41377-023-01079-5. PMC9905593 36750548

[35] H. Gao , X. Jiang , X. Ma , et al., “Bio‐Inspired Mid‐Infrared Neuromorphic Transistors for Dynamic Trajectory Perception Using PdSe_2_/Pentacene Heterostructure,” Nature Communications 16 (2025): 5241, 10.1038/s41467-025-60311-5. PMC12141662 40473631

[36] Z. Han , S. Tian , H. Wang , et al., “Low‐Thermal Budget Fabrication of Two‐Dimensional Schottky Diodes for Broadband Convolutional Processing,” Nano Research 18 (2025): 94907049, 10.26599/NR.2025.94907049.

[37] J. Huang , X. Peng , Y. Wang , et al., “Polarity‐Switchable Logic and In‐Sensor Computing with Gate‐tunable Two‐Dimensional PtTe_2_/WSe_2_ Heterojunctions,” Materials & Design 260 (2025): 115027, 10.1016/j.matdes.2025.115027.

[38] Z. Yang , L. Liao , F. Gong , et al., “WSe_2_/GeSe Heterojunction Photodiode With Giant Gate Tunability,” Nano Energy 49 (2018): 103–108, 10.1016/j.nanoen.2018.04.034.

[39] S. Yuan , P. Han , C. Liu , et al., “Gate‐Programmable Broadband Photodetector for In‐Sensor Computing and Encryption,” Advanced Functional Materials 36 (2026): 75589, 10.1002/adfm.75589.

[40] Y. Yu , Q. Deng , K. Xin , N. Huo , and Z. Wei , “In‐Sensor Polarimetric Optoelectronic Computing Based on Gate‐Tunable 2D Photodetector,” IEEE Electron Device Letters 45 (2024): 645–648, 10.1109/LED.2024.3362827.

[41] G.‐X. Zhang , Z.‐C. Zhang , X.‐D. Chen , et al., “Broadband Sensory Networks With Locally Stored Responsivities for Neuromorphic Machine Vision,” Science Advances 9 (2023): adi5104, 10.1126/sciadv.adi5104. PMC10881039 37713483

[42] Y. Wang , P. Wu , Z. Wang , et al., “Air‐Stable Low‐Symmetry Narrow‐Bandgap 2D Sulfide Niobium for Polarization Photodetection,” Advanced Materials 32 (2020): 2005037, 10.1002/adma.202005037. 32985021

[43] L. Sun , T. Gao , X. Li , et al., “High‐Performance p‐Type Monolayer Tungsten Diselenide Transistors,” Nature Electronics 9 (2026): 758–765, 10.1038/s41928-026-01637-w.

[44] J. Liu , Q. Hao , H. Gan , et al., “Selectively Modulated Photoresponse in Type‐I Heterojunction for Ultrasensitive Self‐Powered Photodetectors,” Laser & Photonics Reviews 16 (2022): 2200338, 10.1002/lpor.202200338.

[45] H. Quan , C. Ma , M. Wu , et al., “Bioinspired Multi‐Dimensional Convolution Kernels Enabled by Gate‐Programmable NiTe_2_/WSe_2_ Nanostrip Photodetector,” Matter 9 (2026): 102751.

[46] T. Bu , X. Duan , C. Liu , et al., “Electrically Dynamic Configurable WSe_2_ Transistor and the Applications in Photodetector,” Advanced Functional Materials 33 (2023): 2305490, 10.1002/adfm.202305490.

[47] Y. Cen , Y. Tu , J. Zhu , Y. Hu , Q. Hao , and W. Zhang , “Photoinduced Contact Evolution and Junction Rearrangement in Two‐Dimensional van der Waals Heterostructure,” Advanced Functional Materials 33 (2023): 2306668, 10.1002/adfm.202306668.

[48] Y. He , X. Mao , Y. Berencén , et al., “Bidirectional Self‐Powered Photoresponse in GeS_x_Se_1‐x_/WSe_2_ van der Waals Heterostructures for Advanced Neuromorphic Vision Systems,” Advanced Functional Materials 36 (2026): 17969, 10.1002/adfm.202517969.

[49] S. Zhang , X. Huang , Y. Chen , et al., “Black Arsenic Phosphorus Mid‐Wave Infrared Barrier Detector With High Detectivity at Room Temperature,” Advanced Materials 36 (2024): 2313134, 10.1002/adma.202313134. 38331419

[50] M. C. Nguyen , V. K. Dat , N. T. Duong , et al., “High–Performance Polarity–Reconfigurable Transistor and Photodiode via Asymmetric Transferred Schottky and Evaporated Ohmic Contacts,” Advanced Functional Materials 36 (2026): 27192, 10.1002/adfm.202527192.

[51] N. T. Duong , Y. Shi , S. Li , et al., “Coupled Ferroelectric‐Photonic Memory in a Retinomorphic Hardware for In‐Sensor Computing,” Advanced Science 11 (2024): 2303447, 10.1002/advs.202303447. 38234245 PMC10966535

[52] J. Liu , H. Gan , Q. Hao , et al., “High‐Performance Visible and Near‐Infrared Dual‐Band Photodetector Based on Anisotropic 2D NbS_3_ With Perpendicularly Reversed Polarization Behaviors,” InfoMat 7 (2025): 12625, 10.1002/inf2.12625.

