# Supplementary material for: Gate‐Tunable Polarity‐Reconfigurable Photovoltaic Response With Dual Linearity in NbS3/WSe2 Heterostructures for In‐Sensor Image Processing
Source: Adv Sci (Weinh). 2026 Sep 6:e77288. Online ahead of print. doi: 10.1002/advs.77288 (PMC13547697; doi:10.1002/advs.77288)
Supplement: Supplementary file 1 [file ADVS-9999-e77288-s001.docx]

**Supporting Information**

**Gate-Tunable Polarity-Reconfigurable Photovoltaic Response with Dual Linearity in NbS_3_/WSe_2_ Heterostructures for In-Sensor Image Processing**

*Jidong Liu, Jianxiong Xie, Qiaoyan Hao, Yutao Hu, Zhen He, Haolin Liu, Danting Lin, Haibo Gan, Yudi Tu, and Wenjing Zhang**

Dr. J.D. Liu, J.X. Xie, Dr. Q.Y. Hao, Y.T. Hu, Z. He, H.L. Liu, D.T. Lin, Dr. H.B. Gan, Dr. Y.D. Tu, Prof. W.J. Zhang
State Key Laboratory of Radio Frequency Heterogeneous Integration,

International Collaborative Laboratory of 2D Materials for Optoelectronics Science and Technology of Ministry of Education,

Institute of Microscale Optoelectronics,

Shenzhen University,

Shenzhen 518060, China
E-mail: wjzhang@szu.edu.cn

**Figure S1**. Representative Raman spectra acquired from the individual NbS_3_ and WSe_2_ flakes and from the overlapped NbS_3_/WSe_2_ heterostructure region.

**Figure S2**. Transfer characteristics of the individual constituent devices. (a) *I*_ds_-*V*_g_ curve of the WSe_2_-based device plotted on a logarithmic current scale at *V*_ds_ = 1 V. (b) *I*_ds_-*V*_g_ curve of the NbS_3_-based device plotted on a logarithmic current scale at *V*_ds_ = 1 V.

**Figure S3**. Morphological characterization of the NbS_3_/WSe_2_ heterostructure. (a) AFM image of the NbS_3_/WSe_2_ heterostructure. (b) Height profiles of the WSe_2_ and NbS_3_ flakes extracted along the white arrows indicated in (a).

**Figure S4**. Schematic energy band profiles of WSe_2_ and NbS_3_ (a) before contact and (b) after contact at *V*_ds_ = 0 V and *V*_g_ = 0 V.

**Figure S5**. Output characteristics of the NbS_3_/WSe_2_ photodetector measured at different incident power densities under illumination at (a) 810 nm, (b) 532 nm, and (c) 450 nm. For each wavelength, the left and right panels correspond to *V*_g_ = 12 V and *V*_g_ = −33 V, respectively.

**Figure S6**. Gate-dependent spatially resolved photocurrent mapping of the NbS_3_/WSe_2_ heterostructure. (a) Optical microscopy image of the NbS_3_/WSe_2_ heterostructure device. The blue, red, black, and yellow dashed lines outline NbS_3_, WSe_2_, graphene, and the metal electrodes, respectively. (b-d) Scanning photocurrent maps measured under 532 nm laser illumination at *V*_ds_ = 0 V and *V*_g_ = 0, 12, and −30 V, respectively.

**Figure S7**. Temporal response of the NbS_3_/WSe_2_ photodetector in the photovoltaic mode under 520 nm illumination. (a) Positive photovoltaic response measured at *V*_ds_ = 0 V, *V*_g_ = 12 V. (b) Negative photovoltaic response measured at *V*_ds_ = 0 V, *V*_g_ = −33 V. The rise/fall time was respectively defined as the time required for the photocurrent to range from 10%/90% to 90%/10% of the peak value.

**Figure S8**. Cyclic endurance of the photoresponse of the NbS_3_/WSe_2_ heterostructure device under illumination at (a,b) 810 nm, (c,d) 532 nm, and (e,f) 450 nm. The positive and negative photovoltaic modes were measured at *V*_g_ = 12 V and *V*_g_ = −33 V, respectively, with *V*_ds_ = 0 V.

**Figure S9**. (a-c) Time-resolved zero-bias photocurrents of a second independently fabricated NbS_3_/WSe_2_ device under 810, 532, and 450 nm illumination at different incident power densities, showing both negative and positive photocurrent states. (d-f) Gate-dependent photovoltaic responses of the same device under 810, 532, and 450 nm illumination, respectively, showing gate-tunable photocurrent polarity switching from negative to positive.

**Figure S10**. Schematic circuit architecture for implementing convolution processing using an array of two-dimensional heterostructure devices. Each electrical symbol represents an independently gated optoelectronic device with a locally tunable polarity-reconfigurable photovoltaic response. The output photocurrent corresponds to the summation of photocurrents generated by the individual devices in the array.

**Figure S11**. Experimentally implemented convolution kernels and corresponding in-sensor image processing results under 532 nm illumination. For each image-processing operation, including sharpness, top Sobel, right Sobel, edge extraction, and emboss, the upper row shows the 3 × 3 gate voltage matrix programmed by *V*_g_, the middle row shows the corresponding short-circuit photoresponsivity (*R*_sc_) kernel, and the bottom row shows the processed image obtained using the experimentally programmed kernel.

**Figure S12**. Experimentally implemented convolution kernels and corresponding in-sensor image processing results under 450 nm illumination. For each image-processing operation, including sharpness, top Sobel, right Sobel, edge extraction, and emboss, the upper row shows the 3 × 3 gate voltage matrix programmed by *V*_g_, the middle row shows the corresponding short-circuit photoresponsivity (*R*_sc_) kernel, and the bottom row shows the processed image obtained using the experimentally programmed kernel.

**Figure S13**. Comparison between standard software convolution kernels and experimentally programmed kernels under 810 nm illumination. The upper panel shows the standard 3 × 3 convolution kernels, including sharpness, top Sobel, right Sobel, edge extraction, and emboss, together with the corresponding simulated images. The lower panel shows the experimentally obtained *R*_sc_ kernels programmed by gate voltage in the NbS_3_/WSe_2_ heterostructure device.

**Figure S14**. (a-c) Gate-dependent external quantum efficiency of the NbS_3_/WSe_2_ heterostructure device under 810, 532, and 450 nm illumination, respectively. (d–f) Corresponding gate-dependent specific detectivity under the three illumination wavelengths.
